# Study on Screening Criteria of Gel-Assisted Polymer and Surfactant Binary Combination Flooding after Water Flooding in Strong Edge Water Reservoirs: A Case of Jidong Oilfield

**DOI:** 10.3390/gels8070436

**Published:** 2022-07-11

**Authors:** Fuquan Luo, Xiao Gu, Wenshuang Geng, Jian Hou, Changcheng Gai

**Affiliations:** 1 School of Petroleum Engineering, China University of Petroleum (East China), Qingdao 266580, China; jxgs_luofq2011@petrochina.com.cn; 2 Research Institute of Exploration and Development, Jidong Oilfield Company, PetroChina, Tangshan 063004, China; kfjs_gxiao@petrochina.com.cn (X.G.); ls_gengwenshuang@petrochina.com.cn (W.G.); ls_gaich@petrochina.com.cn (C.G.)

**Keywords:** strong edge water reservoir, gel-assisted polymer and surfactant flooding, numerical simulation, screening criteria

## Abstract

Strong edge water reservoirs have sufficient natural energy. After long-term natural water flooding development, it is in the stage of ultrahigh water cut. There is an urgent need to change the development mode and improve the development effect. Taking Jidong Oilfield as an example, the mechanism model of strong edge water reservoirs is established by using the method of numerical simulation. Then, the factors and rules affecting the effects of gel-assisted polymer and surfactant binary combination flooding are studied. The screening criteria of gel-assisted polymer and surfactant binary combination flooding in strong edge water reservoirs are obtained. The results show that the existence of edge water is not conducive to binary combination flooding. Smaller water volumetric multiples and larger oil-bearing areas are more suitable for binary combination flooding. Compared with closed reservoirs, binary combination flooding in strong edge water reservoirs is more difficult to establish a displacement pressure gradient. The reservoir with high crude oil viscosity is not suitable for binary combination flooding. Gel-assisted polymer and surfactant binary combination flooding can be adopted for reservoirs with an oil-bearing area greater than 0.2 km^2^, a water volumetric multiple less than 200, and oil viscosity less than 100 mPa·s. The research results are of guiding significance for the reservoir selection of gel-assisted polymer and surfactant binary combination flooding after natural water flooding.

## 1. Introduction

In recent years, oilfields have entered the exploitation period of high water cut or extrahigh water cut, causing the production to decline rapidly. Changing the development mode and pursuing higher recovery are urgent problems to be solved in oilfield development. Chemical flooding, thermal recovery, gas flooding, and microbial flooding are four recognized EOR technologies [1,2,3,4]. Among them, chemical flooding accounts for about a quarter of the world’s total EOR production and is the EOR technology with the largest oil recovery potential in China, which has broad application prospects [5,6,7,8,9]. As an effective method of enhancing oil recovery, chemical flooding has gradually formed a variety of chemical flooding technologies such as polymer flooding and binary or ternary combination flooding. Since the field tests began in the United States in the early 1960s, many laboratory tests and field tests have been carried out by various oil companies, and much work has been conducted on the screening and field applicability of binary combination systems [10,11,12,13,14].

Among these chemical technologies, surfactant-polymer flooding has been widely applied because it includes the mechanisms of improving sweep efficiency, reducing the interfacial tension, and changing the wettability. For instance, Alsofi et al. [15] have conducted a numerical simulation of polymer and surfactant coreflooding by UT-Chem and give the optimal higher polymer: surfactant concentration ratio for carbonates. Rai et al. [16] presented a scaling model that is capable of producing reasonable estimates of oil recovery for a polymer and surfactant flooding. Aramideh et al. [17] used an extensive set of laboratory data including polymer rheology, surfactant phase behavior, polymer permeability reduction, and capillary desaturation along with results from sensitivity analysis to build a mechanistic surfactant-polymer flood model. Yu et al. [18] developed a method to calculate the oil–water proportion in pores during surfactant-polymer flooding. Naik et al. [19] proposed a framework for history matching of surfactant-polymer coreflood process. Liu et al. [20] investigated the effects of pore structure on the enhanced oil recovery of surfactant-polymer flooding to understand the displacement characteristics and the remaining oil-displacement process by the surfactant-polymer flooding in cores with different pore structures. Khormali et al. [21,22] developed a new type of asphaltene removal solvent packs (TPMDS) under static and dynamic conditions through a series of experiments. The core displacement test results showed that with the increase in injection rate, the amount of asphaltene precipitation in the core sample increases. However, the efficiency of the developed solvent does not decrease by changing the injection rate, and the reason is that the performance of TPMDS is improved by the synergistic effect of asphaltene dis-solution. Furthermore, through experimental and simulation methods, they also studied the variation of optimal asphaltene mass concentration under different radial distances, production time, and isotherm types of well spacing to improve reservoir permeability damage. Many scholars studied the factors influencing oil recovery of surfactant-polymer flooding. Tan et al. [23] demonstrated that the decrease in interfacial tension can effectively modify the wettability of reservoir rock. Meanwhile, the sweep efficiency improves with the increase in the viscosity of the SP system. Van et al. [24] studied the effects of impermeable shale barriers on the performances of surfactant flooding and surfactant-polymer (SP) flooding through numerical simulation by CMG (STARS) software. The results show that small-type barriers have a positive effect on surfactant flooding to enhance oil recovery, while barriers of any structure type in SP flooding have an adverse effect. Some scholars [25,26,27,28] demonstrated that high salinity and can reduce the emulsification ability of the surfactant while high temperature can cause the increase in the interfacial tension for polymers, and both high salinity and high temperature can reduce the viscosity of polymer solutions. The above studies focus on the influencing factors of SP flooding in closed reservoirs, but there is no systematic study on strong edge water reservoirs.

It is worth noting that though the profile control ability of SP flooding is not effective, gel-assisted polymer and surfactant flooding seems like a promising technology. Many scholars have found that gel can be used as an effective profile control chemical agent and carried out a variety of research. For example, Deng et al. [29] used nuclear magnetic resonance imaging technology to study the profile control ability of gel. The experimental results showed that the gel can effectively plug the high-permeability area and then the displacing phase can flow into the low-permeability area to displace more oil. Gel has good permeability selectivity, that is, it preferentially enters the large pores with high permeability. At the same time, due to its high strength and deep migration, it can increase the seepage resistance with high permeability, promote the flow diversion, and expand the swept volume of water drive. Di et al. [30] investigated three combination injection patterns with polymer and gel through nuclear magnetic resonance imaging technology. They found that the highest oil displacement efficiency can be obtained by using gel flooding after polymer flooding. Yin et al. [31] developed a new type of in situ gel system and demonstrated that this gel system has good injectivity and can selectively block high-permeability areas during pore-flooding tests. Cui et al. [32] showed that the gel system can displace more oil from small pores through the combination of the coreflooding experiments and microdisplacement experiments. Zhao et al. [33] proposed a new, dispersed particle, gel-strengthened polymer-surfactant. This new system has a higher viscosity and profile control ability than the conventional SP system. Wei et al. [34] carried out parallel core tests and studied the optimize parameters including the injection volume and profile control radius during the injection of the gel system. Chen et al. [35] proposed a mechanistic model about preformed particle gel and surfactant. This model consists of the interaction between preformed particle gel and surfactant and shows that there is a best injection rate to obtain better conformance control. Wang et al. [36] found the gel has more retention and better strength than polymer flooding during coreflooding test, leading to a more effective profile and control ability.

In general, the existing chemical flooding technology is currently mainly used in relatively closed reservoirs with weak natural energy, while the shallow reservoirs in Jidong Oilfield mainly rely on natural energy development, and edge and bottom water is active and the reservoirs are not closed. Whether chemical flooding technology can adapt to this kind of reservoir has not been systematically studied.

In this paper, by using the method of reservoir numerical simulation, the influencing factors of gel-assisted polymers and surfactant flooding in strong edge water reservoirs are studied, and the screening criteria of gel-assisted polymers and surfactant flooding in strong edge water reservoirs are established. The research results provide guidance for the selection of EOR methods in the later stage of similar strong edge water reservoirs.

## 2. Determination of Chemical Agent Properties by Experimental Fitting

The physical properties of chemicals such as surfactant and polymer can be obtained by various methods, such as the atomic simulation and experimental measurements, etc. For the atomic simulation, for instance, Han et al. [37] used the DFTB+ method to calculate the bonding interaction and adsorption structure changes of IM and OP molecules before and after coadsorption on Fe surface. They found that the mixture can change the electron supply of inhibitor molecules with higher E_HoMo_ and lower E_LUMo_, thus enhancing the bonding interaction of both inhibitor agent and inhibitor additive with metal surface. Allce et al. [38] introduced a new DFTB simulation method which can efficiently characterize the physicochemical properties of surfactants. In this paper, the numerical simulation method is used to determine chemical agent properties.

According to the characteristics of Jidong, shallow reservoirs such as a small oil-bearing area and active edge-bottom water, 3D physical simulation experimental devices for the edge water reservoir was developed. The device consists of a model system, a data acquisition and processing system, a production metering system, and an automatic control system. Some water intrusion channels are set outside the 3D model, while the edge water permeation plate is set inside the 3D model, which was composed of filling holes and filter mesh. The edge water is injected by an ISCO pump via the outside water invasion channels, infill holes, and filter mesh. Additionally, all of these can support the experimental research on the law of water flooding and chemical flooding under the multidirectional edge water invasion condition. Through a 3D physical simulation experiment of gel-assisted polymer and surfactant binary combination flooding under three kinds of water energy, namely, no edge water, weak edge water, and strong edge water, the influence mechanism of edge water energy on the development effect of gel-assisted polymer and surfactant binary combination flooding was clarified [39].

On the basis of the 3D experiment, the numerical simulation mechanism model of edge water flooding is established by using CMG numerical simulation software. The mechanism model is completely consistent with the 3D experiment model. The reservoir length, width, and thickness are 30 cm, 30 cm, 15 cm respectively, and the average grid size is 3 × 3 × 3 cm, with a total of 500 grid cells. The mechanism model parameters are shown in Table 1.

**Figure 1:**
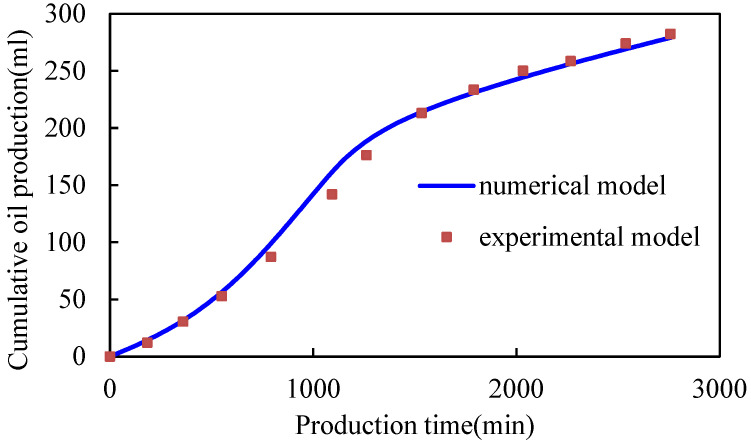
Comparison of experimental and numerical model cumulative oil production.

**Figure 2:**
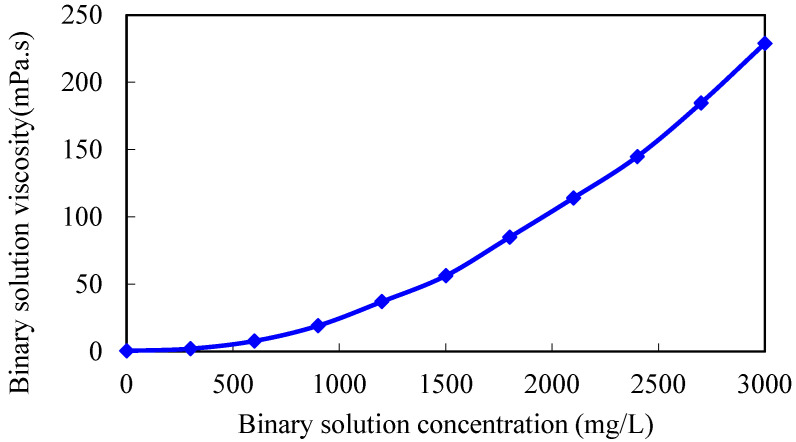
Viscosity–concentration curve of polymer and surfactant binary chemical solution.

**Figure 3:**
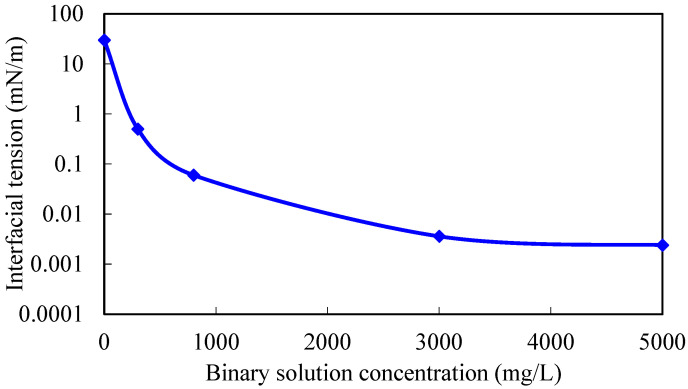
Interfacial tension–concentration curve of polymer and surfactant binary chemical solution.

By modifying the chemical agent parameters in the mechanism model, 3D physical simulation experimental results of gel-assisted polymers and surfactant binary combination flooding are fitted, and the viscosity–concentration curve and the interfacial tension curve of the polymer and surfactant binary chemical solution are obtained. The fitting curve of the experimental cumulative oil is shown in Figure 1, and the fitting accuracy is 98.5%. The residual resistance factor of the gel system is 100. The viscosity–concentration curve and the interfacial tension curve of the polymer and surfactant binary chemical solution are shown in Figure 2 and Figure 3, which can be used for subsequent numerical simulation calculations.

## 3. Simulation Study of Gel-assisted Polymer and Surfactant Flooding in Edge Water Reservoirs

### 3.1. Establishment of Reservoir Numerical Model

**Figure 4:**
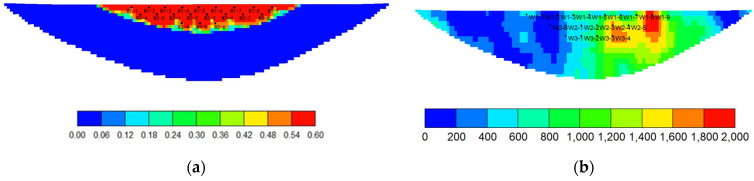
Reservoir model of edge water reservoir. (**a**) Based on initial oil saturation. (**b**) Based on plane permeability distribution.

**Figure 5:**
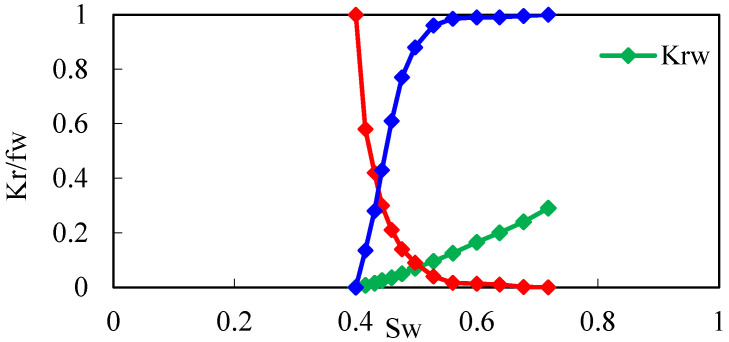
Oil–water phase permeability curve.

Using a CMG simulator, according to the actual reservoir characteristics, the edge-water broken-nose geological model was established with the scale of 20 m × 20 m × 0.5 m, and the grid system was 129 × 27 × 10 with a total of 34,830 grids (Figure 4). The oil-bearing area is 0.3 km^2^, and the outside is edge water with a water volumetric multiple of 200 times. The dip angle of the formation is 5°. The establishment of the basic model takes into account the actual reservoir physical properties, intralayer, and in-plane heterogeneity (Table 2). Plane permeability distribution is shown in Figure 4. The oil–water phase permeability curve is shown in Figure 5.

**Figure 6:**
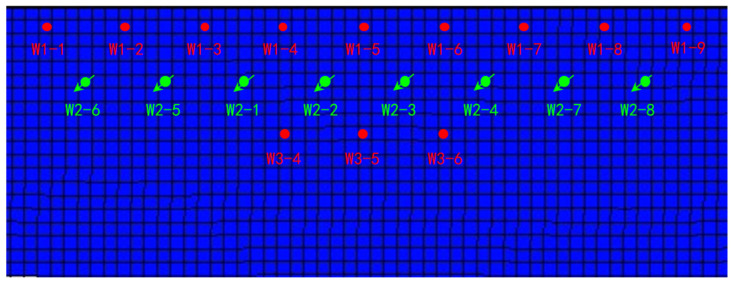
Chemical flooding well pattern of reservoir model.

In the natural water flooding stage, an ir-regular, triangular well pattern is used. Three rows of production wells are deployed along the high part of the fault and the well spacing is 150 m. When the water cut had reached about 98%, the reservoir development mode was changed from edge water flooding to gel-assisted polymer and surfactant binary combination flooding. The staggered-row well pattern was used to convert the second row of oil wells to injection wells (Figure 6). The concentrations of injected surfactant and polymer solution were 3000 mg/L and 500 mg/L. The ratio of polymer to crosslinking agent in the gel system is 1:1, and the concentration is 500 mg/L. The injection volume of the chemical displacement system is 0.6 PV. Based on the plane permeability distribution, the dominant seepage channel volume is evaluated, and the gel injection volume is determined. Gel is 0.05 PV and surfactant-polymer solution is 0.55 PV. The injection rate is 0.1 PV per year (0.1 PV/a), equivalent to 10 m^3^/d for a single well.

### 3.2. Simulation Analysis of Influencing Factors

Based on the numerical simulation method, the factors influencing oil recovery of gel-assisted surfactant-polymer flooding are studied. Some factors are related to the oil properties, which are described separately for different oil properties, such as injection rate and viscosity ratio between displacement phase and crude oil.

#### 3.2.1. Water Volumetric Multiple

The water volumetric multiple is the ratio of the edge water pore volume to the crude oil pore volume. The larger the water volumetric multiple is, the more active the edge water is. When the water volumetric multiple is greater than or equal to 100 times, the edge water energy is considered to be strong. In order to compare the concentration difference of chemical agent front under different edge water energies, the concept of dilution factor is defined as the ratio of chemical agent concentration between no edge water and strong edge water, which characterizes the dilution degree of edge water to chemical agent.

**Figure 7:**
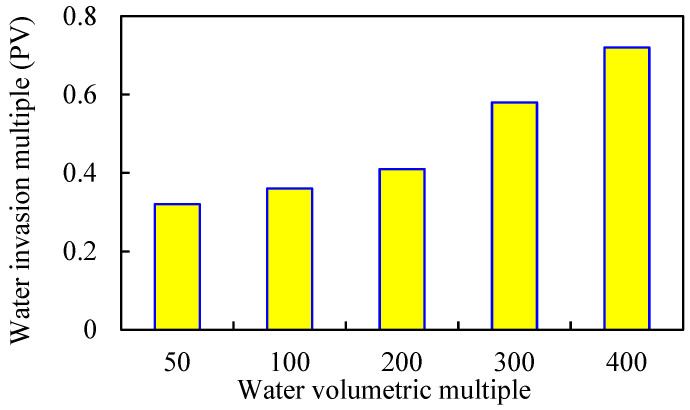
Water invasion multiple under different water volumetric multiples.

**Figure 8:**
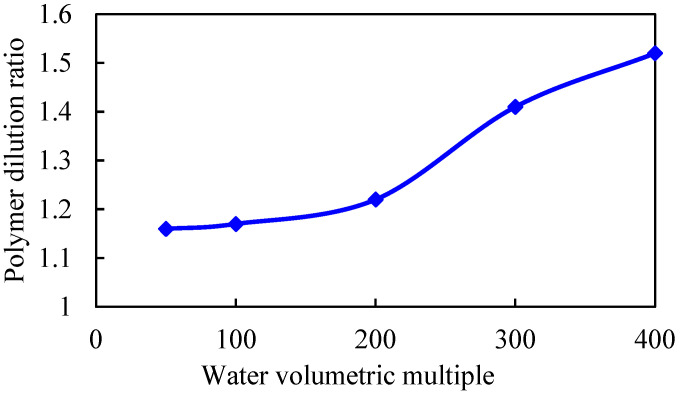
Chemical agent dilution ratio curve in different water volumetric multiples.

**Figure 9:**
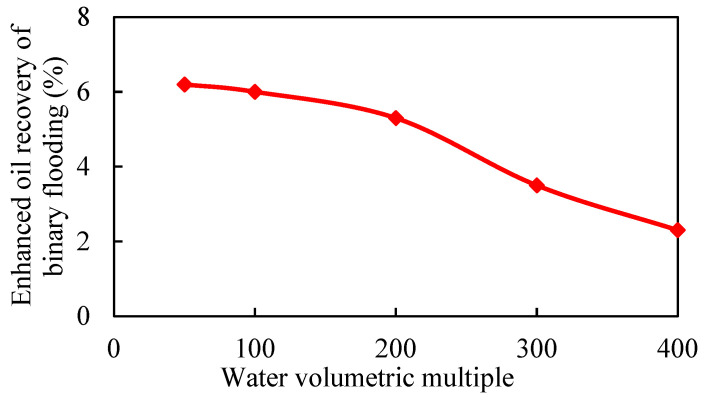
Variation curve of enhanced oil recovery in different water volumetric multiples.

**Figure 10:**
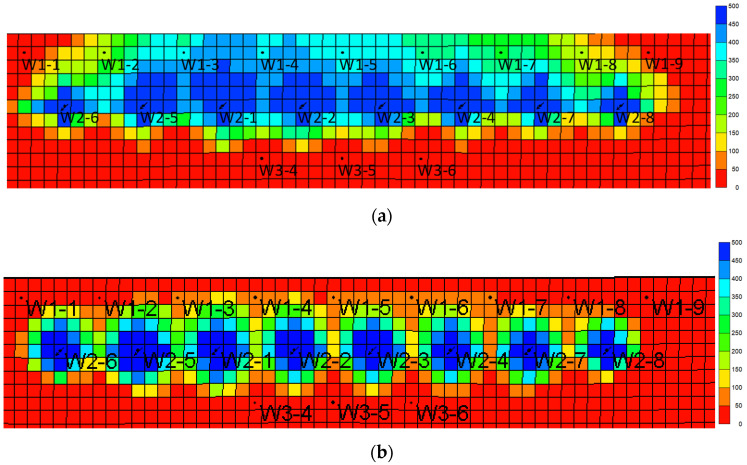
Distribution of polymer concentration under different water volumetric multiples. (**a**) Polymer concentration with the water volumetric multiple of 50. (**b**) Polymer concentration with the water volumetric multiple of 200.

Under the condition that other parameters remain unchanged, the binary displacement process is set with the injection volume of 0.6 PV and the injection rate of 0.1 PV/a. The model adopts different water volumetric multiples, which are 50, 100, 200, 300, and 400 times, respectively. The simulation calculation results are shown in Figure 7, Figure 8, Figure 9 and Figure 10.

As the water volumetric multiple is larger, the water invasion during the development process of the gel-assisted polymer and surfactant binary combination flooding is larger, the dilution ratio of the chemical agent is larger, and the enhanced oil recovery rate is lower. Compared with 50 times of weak edge water condition, the loss rate of enhanced oil recovery under 400 times of water body condition reaches 63%. When the water volumetric multiple is greater than 200 times, the polymer concentration decreases significantly, resulting in a significant decrease in the enhanced oil recovery.

#### 3.2.2. Oil-Bearing Area

**Figure 11:**
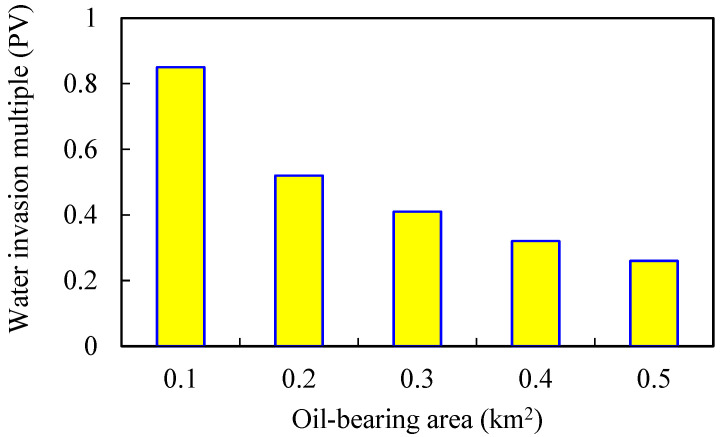
Water invasion multiple under different oil-bearing areas.

**Figure 12:**
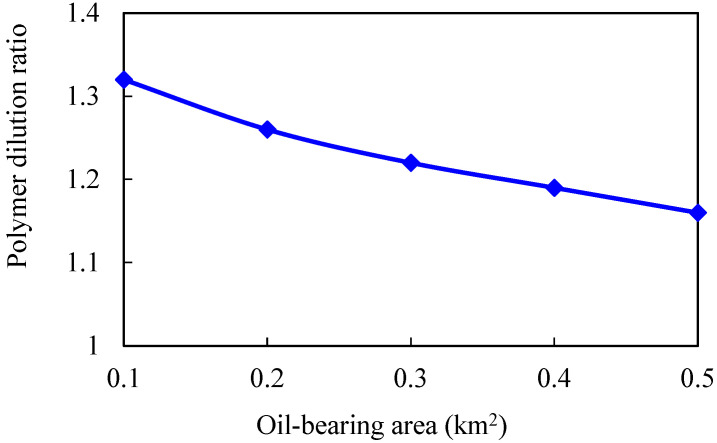
Chemical agent dilution ratio curve in different water volumetric multiples.

**Figure 13:**
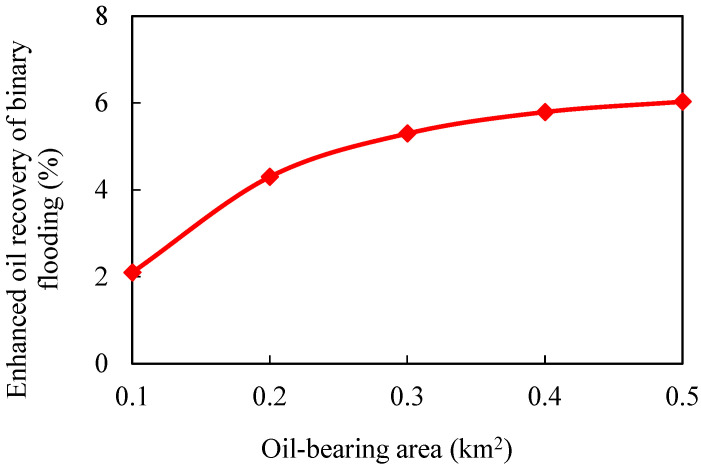
Variation curve of enhanced oil recovery in different water volumetric multiples.

**Figure 14:**
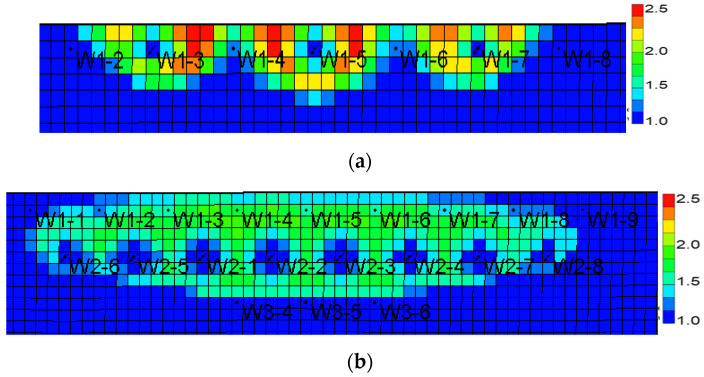
Polymer concentration dilution multiple under different oil-bearing areas. (**a**) Dilution ratio of polymer concentration with oil-bearing area of 0.1 km^2^. (**b**) Dilution ratio of polymer concentration with oil-bearing area of 0.3 km^2^.

Keeping other parameters unchanged, numerical models of different oil-bearing areas are established. The oil-bearing areas are 0.1, 0.2, 0.3, 0.4, and 0.5 km^2^, respectively. The simulation results are shown in Figure 11, Figure 12, Figure 13 and Figure 14.

The larger the oil-bearing area is, the more perfect the displacement well pattern is. When the oil-bearing area is 0.1 km^2^, the well row is a row, so the regular area well pattern cannot be formed. Moreover, the oil well is close to the water boundary, which means that the water invasion multiple is large, and the polymer solubility will be significantly diluted. With the increase in oil-bearing area, water influx and dilution ratio of chemical agent in binary flooding stage gradually decrease, while enhanced oil recovery gradually increases. When the oil-bearing area is greater than 0.2 km^2^, the increase in enhanced oil recovery slows down.

#### 3.2.3. Injection–Production Well Pattern

**Figure 15:**
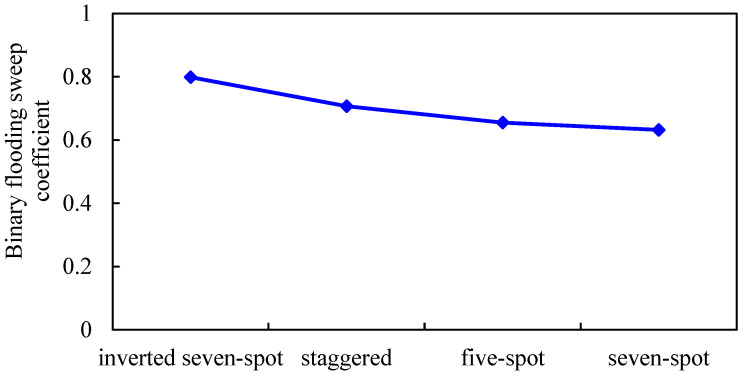
Sweep coefficient of different well patterns.

**Figure 16:**
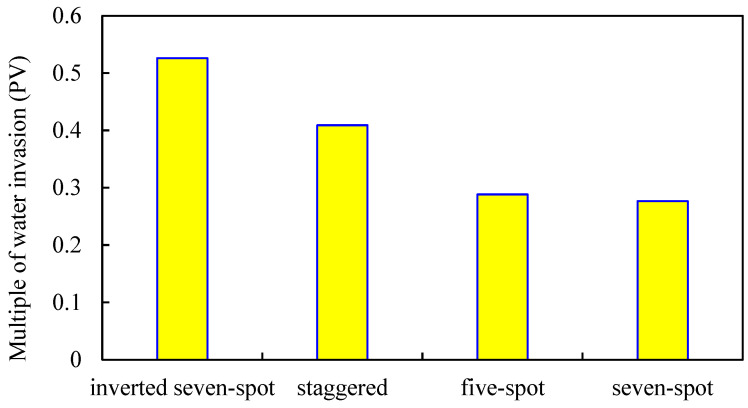
Water invasion of different well patterns.

**Figure 17:**
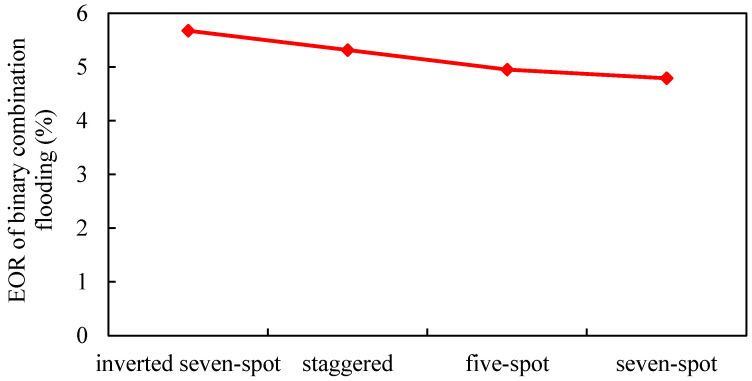
EOR of different well patterns.

**Figure 18:**
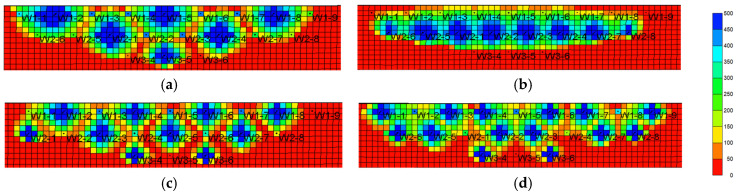
Polymer distribution of different well patterns. (**a**) Inverted seven-spot (injector–producer ratio 6:14). (**b**) Staggered (injector–producer ratio 8:12). (**c**) Five-spot (injector–producer ratio 10:10). (**d**) Seven-spot (injector–producer ratio 14:6).

The oil-bearing area is 0.3 km^2^, and the inverted seven-spot well pattern, staggered well pattern, seven-spot well pattern, and five-spot well pattern are designed (other parameters remain unchanged). The influence of different well patterns on the development effect of gel-assisted polymer and surfactant binary flooding is studied. The results are shown in Figure 15, Figure 16, Figure 17 and Figure 18.

The sweep coefficient of the inverted seven-spot well pattern is the largest, so the binary combination flooding effect is the best [40]. The staggered well pattern is the second and the seven-spot pattern is the worst. Compared with the inverted seven-spot pattern, the enhanced oil recovery and sweep coefficient of the seven-spot pattern decreased by 15.1% and 20.7%, respectively, and the decline rate of enhanced oil recovery is smaller than that of the sweep coefficient. The main reason is that from the inverted seven-spot well pattern to the seven-spot well pattern, the injection–production well ratio gradually increases and the invasion of edge water gradually decreases.

#### 3.2.4. Oil Viscosity

**Figure 19:**
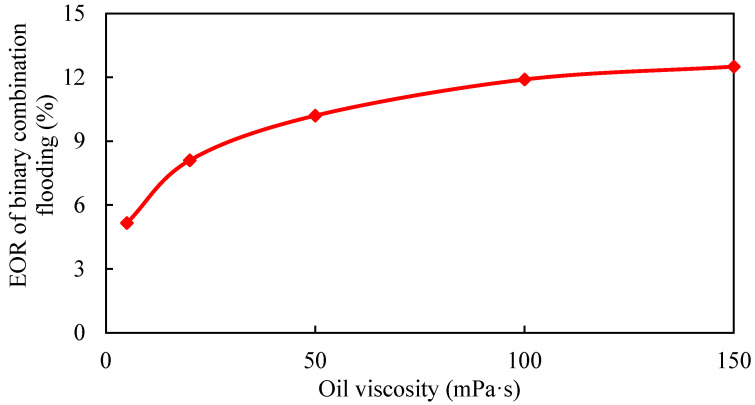
EOR of binary combination flooding with different oil viscosity.

The viscosity of crude oil has a great influence on the development effect of water flooding and chemical flooding [41,42]. The viscosity of crude oil at 5, 20, 50, 100, and 150 mPa·s was simulated by single factor analysis (keeping other parameters unchanged and surfactant-polymer solution viscosity equal to crude oil). The results are shown in Figure 19 and Table 3.

With the increase in oil viscosity, the difference in the oil–water mobility ratio becomes larger, the oil recovery decreases in the edge water flooding stage, and the remaining oil after the edge water flooding stage is large. The gel-assisted polymer and surfactant binary flooding can improve the oil–water mobility ratio, expand sweep volume, and enhance oil recovery. Therefore, with the increase in oil viscosity, the enhanced oil recovery of gel-assisted polymer and surfactant binary flooding becomes larger. However, when the oil viscosity is greater than 100 mPa·s, the mobility control ability of gel-assisted polymer and surfactant binary flooding system decreases, and increase in EOR slows down.

#### 3.2.5. Injection Rate

**Figure 20:**
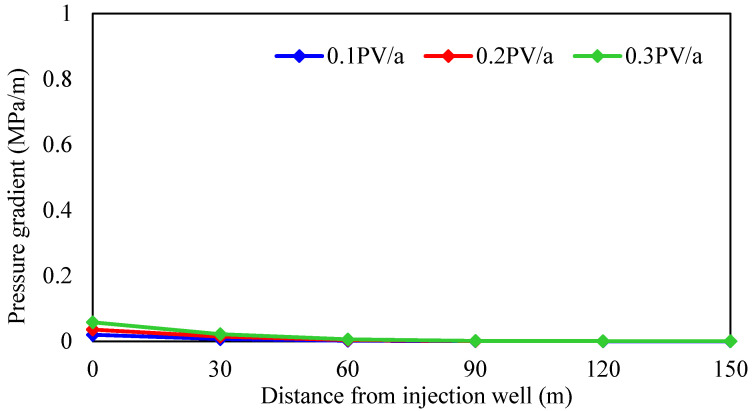
Relationship between injection rates and pressure gradient in thin-oil reservoir.

**Figure 21:**
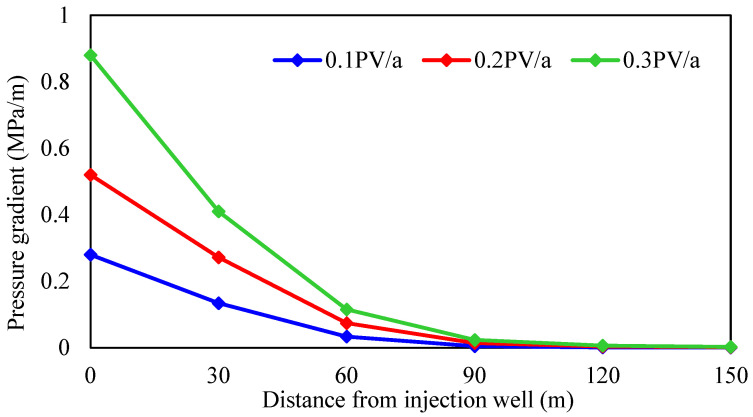
Relationship between injection rates and pressure gradient in heavy-oil reservoir.

**Figure 22:**
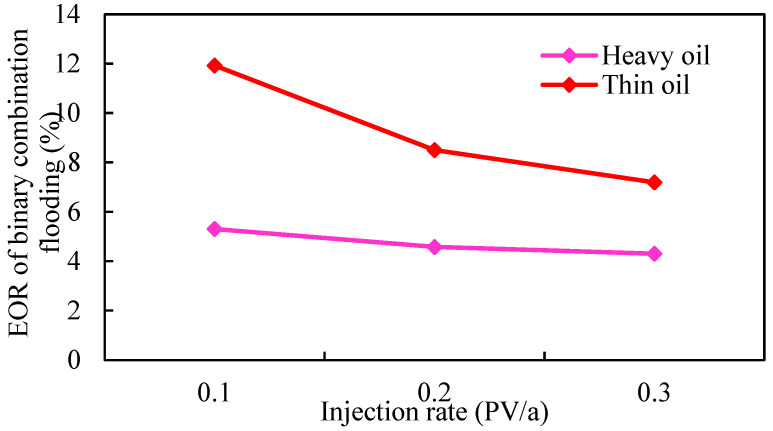
Relationship between injection rate and enhanced oil recovery.

According to different crude oil properties, the reservoir is divided into thin oil and heavy oil, and the influence of the injection rate on the development effect of gel-assisted polymer and surfactant binary flooding is studied. The injection rate is 0.1 PV/a, 0.2 PV/a, and 0.3 PV/a, the oil viscosity in thin-oil reservoirs is 5 mPa·s, and the oil viscosity in heavy-oil reservoirs is 100 mPa·s. The simulation results are shown in Figure 20, Figure 21 and Figure 22 and Table 4.

As shown in Figure 20 and Figure 21, the displacement pressure gradient required of heavy-oil reservoirs is much larger than that of thin-oil reservoirs due to the high seepage resistance. Due to the external strong edge water supply, there is no pressure drop funnel at the bottom of the production well, and the pressure drop funnel only exists near the injection well. At different injection rates, the pressure gradient near the injection well changes significantly, while the pressure gradient near the production well has little difference. Therefore, the reservoir displacement pressure gradient can only be established by increasing the formation pressure near the injection well, and it is more difficult to establish the displacement pressure gradient compared with the closed reservoir. Considering the upper limit of injection pressure, wellbore additional pressure, and reservoir fracture pressure, the maximum bottom hole flow pressure of injection well is 50 MPa in simulation.

As shown in Figure 22 and Table 3, injection rate has little effect on the enhanced oil recovery of gel-assisted polymer and surfactant binary flooding in the thin-oil reservoir, but has an obvious effect on the heavy-oil reservoir. The higher the injection rate, the lower enhanced oil recovery. The main reason is that with the increase in injection rate, the required displacement pressure gradient is larger, and the formation pressure gradient is established only near the injection well, which leads to exceeding the upper limit of injection pressure near the injection well, which means that the effective chemical flooding cannot be established. As a result, the injection–production ratio decreases and the effect deteriorates.

#### 3.2.6. Viscosity Ratio between Binary Flooding Solution and Crude Oil

**Figure 23:**
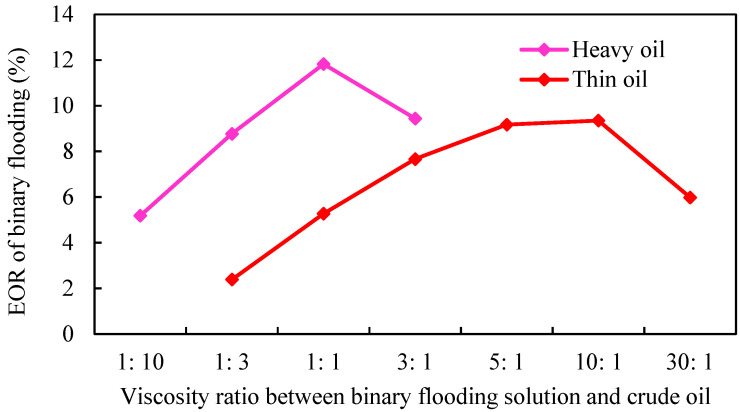
Relationship between EOR and viscosity ratio.

According to different crude oil properties, the reservoir is divided into thin oil and heavy oil. The effects of viscosity ratio between binary flooding solution and crude oil on the development of gel-assisted polymer and surfactant binary flooding are studied. The simulation results are shown in Figure 23.

As shown in Figure 23, with the increase in the viscosity of the binary combination system, the enhanced oil recovery of binary flooding increased first and then decreased. The main reason is that the greater the viscosity ratio of displacement phase to crude oil, the greater the displacement pressure gradient. Due to the existence of external edge water energy supply, it is more difficult to establish the displacement pressure gradient in strong edge water reservoirs, which leads to the inability to establish effective chemical displacement, the decline of the injection–production ratio, and a poor displacement effect. The flow resistance of thin-oil reservoir is small, and it is easier to establish effective displacement. The optimal viscosity ratio between binary combination solution and crude oil is 5: 1. Additionally, the optimal viscosity ratio of the heavy-oil reservoir is 1:1, which is lower than that of the thin-oil reservoir.

## 4. Establishment of Screening Criteria

By analyzing the relationship between the various influencing factors and recovery, the inflection points of the increase in recovery was found, and the screening criteria was established. As shown in Table 5, for strong edge water reservoirs, the greater the water volumetric multiple, the worse the gel-assisted polymer and surfactant binary flooding effect. Furthermore, when the water volumetric multiple is more than 200 times, the enhanced oil recovery decreases obviously. When the oil-bearing area is less than 0.2 km^2^, it is not recommended to use gel-assisted polymers and surfactant binary flooding. The viscosity of crude oil is greater than 100 mPa·s, the enhanced oil recovery becomes slow, and the flow control of binary combination system is limited. At the same time, due to the external strong edge water energy supply, the displacement pressure gradient distribution between injection and production wells is different from that of closed reservoirs, which makes it more difficult to establish the displacement pressure gradient, so the viscosity of crude oil should not be too high.

The chemical agent sweep coefficient of the inverted seven-spot well pattern is the largest, and the displacement effect is the best. For thin-oil reservoirs, the optimal viscosity ratio between binary combination solution and crude oil is 5:1, and the injection rate has little effect. For heavy-oil reservoirs, the optimal viscosity ratio between binary combination solution and crude oil is 1:1, and the injection rate is 0.1 PV/a.

## 5. Conclusions

The dilution of multiple chemical agents is defined, which can quantitatively evaluate the distribution difference of the chemical agent concentration field under different water energy levels and reveal the influence mechanism of edge water on chemical flooding effects. Strong edge water has a disadvantageous influence on chemical flooding development. In the process of gel-assisted polymer and surfactant binary flooding development, edge water invasion into the reservoir leads to the decrease in chemical agent concentration. The dilution multiple is 1.52 and the loss rate of enhanced oil recovery is 63%. At the same time, due to the energy supply of strong edge water, there is no obvious pressure drop funnel near the production well, which makes it more difficult for strong edge water reservoirs to establish the displacement pressure gradient, and the boundary of reservoir parameters applicable to chemical flooding is more stringent. Through numerical simulation, the screening criteria of gel-assisted polymer and surfactant binary combination flooding in strong edge water reservoirs are established: oil-bearing area is greater than 0.2 km^2^, water volumetric multiple is less than 200 times, and crude oil viscosity is less than 100 mPa·s.

## Figures and Tables

**Table 1:** Mechanism model parameters of edge water reservoir.

Parameter	Value
Model size (cm)	30 × 30 × 15
Porosity	0.32
Permeability (mD)	2000
Oil viscosity (cp)	5
Water viscosity (cp)	0.45
Initial oil saturation (%)	85
Residual oil saturation (%)	30

**Table 2:** Reservoir model parameters of edge water reservoir.

Parameter	Value
Oil-bearing area (km^2^)	0.3
Reservoir thickness (m)	5
Porosity	0.32
Permeability (mD)	2000
Oil viscosity (cp)	5
Residual oil saturation (%)	28.2
Ir-reducible water saturation (%)	40
Reservoir pressure (MPa)	18
Reservoir temperature (°C)	65
Intralayer variation coefficient	0.7
Plane variation coefficient	0.6

**Table 3:** Recovery of edge water flooding and gel-assisted polymer and surfactant binary flooding.

Viscosity (mPa·s)		Recovery (%)		
Surfactant-Polymer Solution	Crude Oil	Edge Water Flooding	Binary Combination Flooding	Total
5	5	43.8	5.2	49.0
20	20	35.1	8.1	43.2
50	50	29.8	10.2	40.0
100	100	25.6	11.9	37.5
150	150	22.8	12.5	35.3

**Table 4:** Relationship between injection rate and bottom hole flowing pressure.

Injection Rate (PV/a)	Bottom Hole Flowing Pressure of Injection Well (MPa)	
	Thin-Oil Reservoir (5 mPa·s)	Heavy-Oil Reservoir (5 mPa·s)
0.1	15.37	46.92
0.2	16.75	77.85
0.3	18.12	108.77

**Table 5:** Screening criteria for gel-assisted SP flooding in strong edge water reservoirs.

Influence Factors	Limit Value
Water volumetric multiple	≤200 times
Oil-bearing area	≥0.2 km^2^
Oil viscosity	≤100 mPa·s

## Data Availability

Not applicable.
